# Microstructural deformation process of shock-compressed polycrystalline aluminum

**DOI:** 10.1038/s41598-019-43876-2

**Published:** 2019-05-20

**Authors:** Kouhei Ichiyanagi, Sota Takagi, Nobuaki Kawai, Ryo Fukaya, Shunsuke Nozawa, Kazutaka G. Nakamura, Klaus-Dieter Liss, Masao Kimura, Shin-ichi Adachi

**Affiliations:** 1 Division of Biophysics, Department of Physiology, Jichi Medical University, 3311-1 Yakushiji, Shimotsuke, Tochigi 329-0498 Japan; 2 Photon Factory, Institute of Materials Structure Science, High Energy Accelerator Research Organization, 1-1 Oho, Tsukuba, Ibaraki 305-0801 Japan; 3 Division of Earth Evolution Science, Graduate School of Life and Environmental Sciences, University of Tsukuba, 1-1-1 Tennodai, Tsukuba, Ibaraki 305-8572 Japan; 4 Institute of Pulsed Power Science, Kumamoto University, 2-39-1 Kurokami, Kumamoto, 860-8555 Japan; 5 Laboratory for Materials and Structures, Institute of Innovative Research, Tokyo Institute of Technology, R3-10, 4259 Nagatsuta, Yokohama, Kanagawa 226-8503 Japan; 6 Materials Science and Engineering Program, Guangdong Technion- Israel Institute of Technology, 241 Daxue Road, Jinping District, Shantou, Guangdong 515063 China; 7 Technion - Israel Institute of Technology, Haifa, 32000 Israel

**Keywords:** Mechanical properties, Metals and alloys

## Abstract

Plastic deformation of polycrystalline materials under shock wave loading is a critical characteristic in material science and engineering. However, owing to the nanosecond time scale of the shock-induced deformation process, we currently have a poor mechanistic understanding of the structural changes from atomic scale to mesoscale. Here, we observed the dynamic grain refinement of polycrystalline aluminum foil under laser-driven shock wave loading using time-resolved X-ray diffraction. Diffraction spots on the Debye-Scherrer ring from micrometer-sized aluminum grains appeared and disappeared irregularly, and were shifted and broadened as a result of laser-induced shock wave loading. Behind the front of shock wave, large grains in aluminum foil were deformed, and subsequently exhibited grain rotation and a reduction in size. The width distribution of the diffraction spots broadened because of shock-induced grain refinement and microstrain in each grain. We performed quantitative analysis of the inhomogeneous lattice strain and grain size in the shocked polycrysalline aluminum using the Williamson-Hall method and determined the dislocation density under shock wave loading.

## Introduction

Microstructural deformation, and grain refinement and rotation in polycrystalline metal materials (for example, engineering alloys and ceramics) under shock compression are important characteristics, which determine material properties, such as strength and elastic-plastic deformation. Shock-induced plastic deformation and shock wave propagation in polycrystalline materials at the grain level (mesoscale) are fundamentally different from those behaviors in single-crystals and amorphous material^1–5^. Specifically, at the mesoscale, cell size-dependent features emerge, for example, particle velocity fluctuations, and rotational deformation fields. Additionally, a hierarchy of dissipative structures form, such as shear bands and heterogeneously distributed defects. Furthermore, a material is undergoing a plastic flow, the shock wave can rapidly generate dislocation slips or twinning in each grain at high strain rates (>10^6^ s^−1^), whereby the dislocation density increases with strain rate, as expressed by Orowan’s law^6–8^. Laser-induced shock wave have been used for grain refinement in peening techniques, such as laser peening, for modifying surface strength, fatigue resistance, and surface feature of materials^9–13^. However, we lack a comprehensive understanding of deformation process at high strain rates. The mechanical behavior of face-centered cubic (fcc) aluminum under shock wave loading has been widely studied with the velocity interferometer system for any reflector (VISAR), which measures the displacement and thus velocity of the reflecting back surface of the sample^14–16^. To date, microstructural deformations under shock wave loading have mainly been studied in post-shock recovery experiments^7,8^. Molecular dynamics (MD) simulation of large-scale shock compression have been performed and the topologies of shock-compressed structure compares well with observations in transmission electron microscopy (TEM) studies of shock-induced plasticity in samples^7,8,17,18^. However, the dislocation density in simulations is typically greater than that in experimental observations of recovered samples. Although the grain deformation of polycrystalline materials under plastic shock flow occurs through the nucleation and movement of dislocations behind the shock wave front, plastic deformation has been primarily studied using the final shock recovery products. The post-shocked structure might have thermally recovered, and is influenced by the shock wave reflection and the residual high temperature after release of the shock pressure. Recently, the slip process of shock-compressed solid materials has been observed by Laue diffraction with broadband X-rays^19–24^ and an X-ray free electron laser^25^. In the plastic deformation of polycrystalline copper under compression at low strain rate, the initially large grains break down and rotate, which is inherent to plastic deformation^26–29^. The application of stroboscopic time-resolved X-ray diffraction using the intense, broadband X-ray pulses enables *in situ* characterization of plastic deformation in shock-compressed polycrystalline materials.

In this paper, we quantitatively analyze the shock-compressed microstructure deformation of polycrystalline aluminum using time-resolved X-ray diffraction. Because of the broad bandwidth energy (Δ*E*/*E* = 1.45%) of the X-ray pulse, we were able to observe the dynamic process of grain refinement and fragmentation in the shock-compressed materials from the X-ray diffraction patterns. Furthermore, we observed the evolution and arrangement of diffraction spots on Debye-Scherrer rings *in situ*. These features derive from individual crystallites and enabled us to determine the *in situ* dislocation density.

## Results

**Figure 1:**
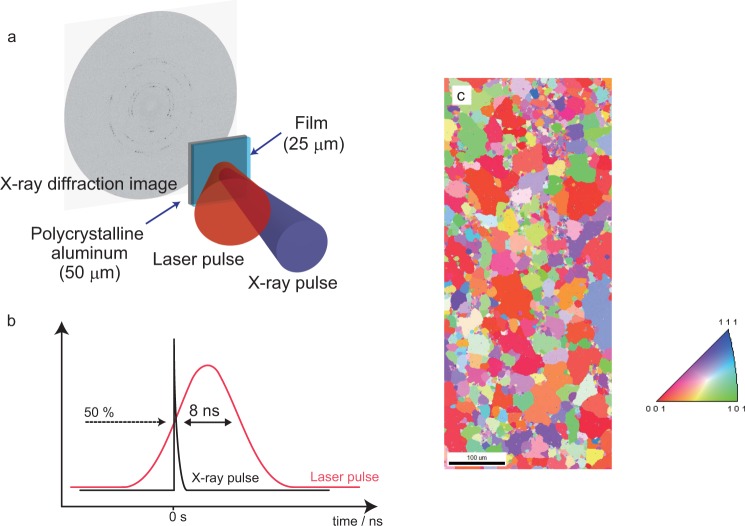
Time-resolved X-ray diffraction and microstructure of polycrystalline aluminum. (**a**) Plasma-confined sample of a polyethylene terephthalateT ablator film (25-*μm* thick) and polycrystalline aluminum foil (50 *μ*m thick), and set-up of the single-shot time-resolved pink X-ray diffraction in the transmission X-ray diffraction geometry. (**b**) Timing chart of the synchronization system between the laser and X-ray pulses. The pulse width of the X-ray and laser are 100 ps and 8 ns, respectively. The X-ray pulse is initiated at *t* = 0 ns when the laser pulse reaches 50% intensity of its maximum intensity. (**c**) Orientation color-coded electron back-scatter diffraction map (vertical direction of the map projected in the standard triangle) of the initial microstructure of polycrystalline aluminum.

We performed single-shot, time-resolved, broad-bandwidth X-ray diffraction using the NW14A beamline at the Photon Factory Advanced Ring of the High Energy Accelerator Research Organization^19,30–32^. The experimental setup is shown in Fig. 1a. The sample target was composed of two layers: a polycrystalline aluminum film and a polymer film for the ablator. The aluminum used in this study was 99.9% pure aluminum foil (Nilaco, Co., Japan), which was produced by an ordinary sheet rolling; the target size was 5 × 5 × 0.05 mm^3^. The cover film was used for the laser ablation plasma was 25 *μm*-thick polyethylene terephthalate. The polycrystalline aluminum foil specimen was coated with a plastic film to produce the laser ablation plasma plume for generating the shock wave into the aluminum foil. The interval between the x-ray pulse and laser pulse at 0 ns on the sample surface was adjusted to 50% of InGaAs photodiode with 80 ps time resolution. Figure 1b shows the timing chart of the X-ray pulse (100 ps) and laser pulse (8 ns).

**Figure 2:**
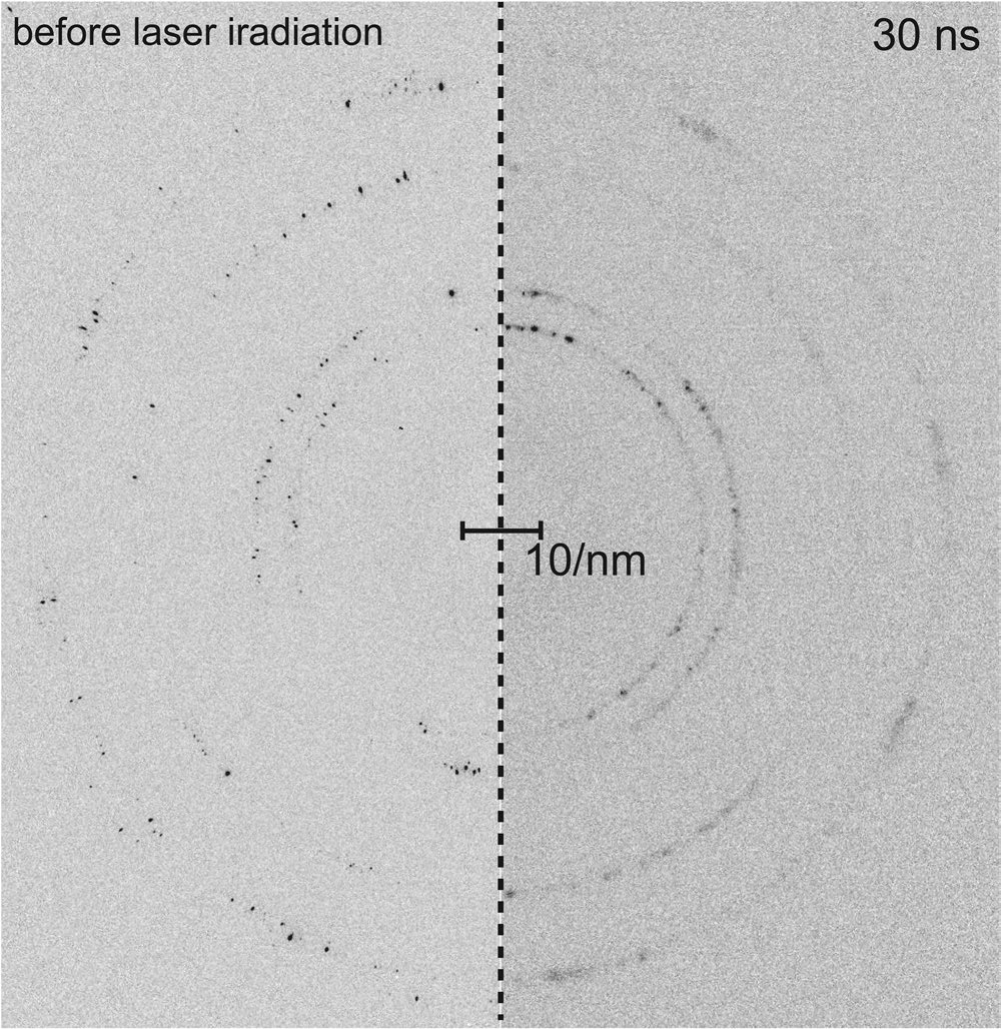
Two-dimensional diffractogram of polycrystalline aluminum. Diffraction images of polycrystalline aluminum foil before laser irradiation (left image) and at 30 ns (right image). Debye-Scherrer rings of the sample are from the 111, 200, 220, 311, and 222 reflections. After laser-induced shock wave loading, the Debye-Scherrer ring was smoothed by shock-induced plastic deformation.

The microstructures of polycrystalline aluminum foil is shown in Fig. 1c, from which initial grain orientation and grain size can be determined. The grain size was distributed from several tens of micrometers to approximately hundred micrometers. The powder diffraction data were obtained from the samples in transmission X-ray geometry. Typical Debye-Scherrer rings from the polycrystalline aluminum foil at ambient pressure and 30 ns after laser irradiation are presented in Fig. 2. Small diffraction spots originating from individual large grains inside the polycrystalline aluminum (as shown in Fig. 1c), were present on the diffraction rings. The Debye-Scherrer ring was composed of 111, 200, 220, and 311 of aluminum. The features in the Debye-Scherrer rings in the azimuthal-angle direction become significantly smoother after shock wave propagation at 30 ns. The number of diffraction spots and broadening increased as a results of laser-shock fragmentation, which causes the formation of dislocation networks, subgrain, intergranular stress and grain fracture.

**Figure 3:**
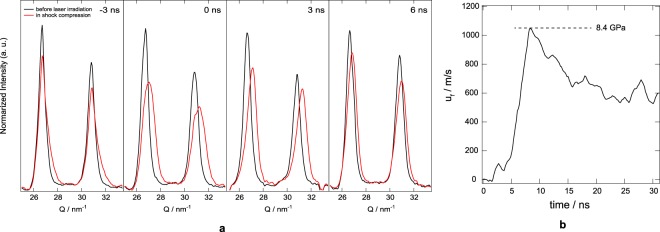
Estimation of shock pressure from the strain. (**a**) X-ray diffraction profiles of the 111 and 200 aluminum peaks at ambient pressure (black curves) and at delay times of −3, 0, 3 and 6 ns (red curves). The maximum peak shift is at 3 ns with a peak lattice strain $\epsilon$ = −0.02. These diffraction profiles were averaged from 25 diffraction images taken from different targets. (**b**) Example Velocimetery interferometer systems for any reflector (VISAR) trace for the same experimental conditions as the time-resolved X-ray diffraction measurement. This velocity profile is from the free-surface of the polycrystalline aluminum film.

During shock compression, the average shock pressure in the polycrystalline aluminum foil was estimated from the average radial diffraction peak shift, Δ*G*, where the lattice strain is given by $\epsilon$ = −Δ*G*/*G* and the volumetric strain is Δ*V*/*V* = 3$\epsilon$ under isotropic and hydrostatic conditions in a given grain. The integrated profiles in the azimuthal angle comprising the aluminum 111 and 200 reflections are shown in Fig. 3a as a function of delay time. To accurately determine the average peak shift and shape, 25 diffraction images were taken at different samples at each delay time. At *t* = −3 ns, the compressed bulk region begins to increase while the shock wave propagates from the aluminum surface. Because of the choice of *t* = 0, the laser pulse front-tail reaches the sample at this negative delay time. The physical starting time *t*_0_, at which the shock wave is generated at the sample surface, is at about −6 ns. At *t*_*max*_ = 3 ns, the entire probed region is maximally compressed by shock wave loading, after which the pressure begins to release because the shock wave has reached the back surface of the sample and is being reflected into a rarefaction wave. From *t*_*max*_ = 3 ns, we calculated the average shock pressure in the compressed region, *P*_*H*_, the shock wave velocity, *U*_*s*_, and the particle velocity, *u*_*p*_, from the shift of the 111 peak and from the Hugoniot equation of state of aluminum. The shocked pressure from the Hugoniot relationship is given by

1 $${P}_{H}=\frac{{\rho }_{0}{C}_{0}^{2}\eta }{{(1-s\eta )}^{2}}$$

where ${\rho }_{0}$ denotes the initial density of aluminum, *C*_0_ is the velocity of sound in aluminum, *s* is the shock data parameter of aluminum, and $\eta$ is the compressive volumetric strain. The value of $\eta$ is found by

2 $$\eta =\frac{V-{V}_{0}}{{V}_{0}}=\frac{{\rm{\Delta }}V}{{V}_{0}}=-\,3\epsilon =3\frac{{\rm{\Delta }}G}{G}.$$

There is a linear relationship over the Hugoniot elastic limit between the shock wave velocity and the particle velocity such that ${U}_{s}={C}_{0}+s{u}_{p}$, where the values for *C*_0_ (5.386 km/s) and *s* (1.339) correspond were the same as those used in ref.^33^. Therefore, the average shock pressure in the entire region compressed by the shock wave was estimated to be 5.43 GPa at 3 ns. After 3 ns, the shock pressure released to ambient pressure. The estimated peak pressure of 5.43 GPa at *t* = 3 ns greatly exceeds the Hugoniot elastic limit of commercial pure aluminum with 50 *μm* thickness (about 1 GPa)^34,35^. Figure 3b shows the VISAR trace for the the same sample and laser energy; the free surface velocity of the polycrysalline aluminum corresponds to the rise time of the laser pulse shape. The peak pressure of pressure profile was 8.4 GPa. At 3 ns delay times, the shock wave close to the free surface, and the pressure profile can be deduced by the VISAR data, it was a 8.4 GPa peak pressure of shock wave followed by a rarefaction wave, and the average pressure was consistent with the results which estimated from the X-ray diffraction.

**Figure 4:**
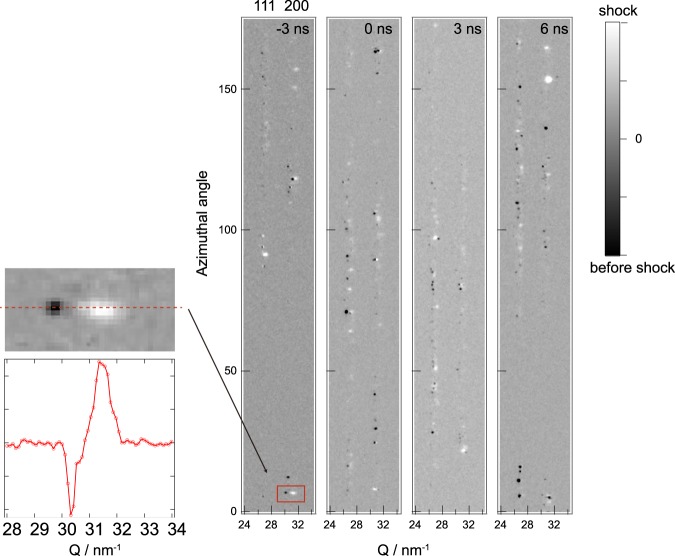
Microstructural deformation behind the shock wave front. Differential intensity from the Debye-Scherrer ring at −3, 0, 3 and 6 ns straightened into azimuthal-angle (that is, scattering-vector) plots. (Inset) At −3 ns, a few Bragg diffraction spots from just behind the shock wave front shift to the high *Q* region, which corresponds to the radial direction.

The diffraction peak widths of each diffraction spot represent the mosaicity of the reflecting grains during and after shock wave-induced elastic-plastic deformation. To display the spottiness in the diffraction pattern as a function of delay time, the differential aluminum 111 and 200 Debye-Scherrer rings were straightened to the azimuthal angle and are shown in Fig. 4. The diffraction images before laser irradiation were subtracted from those of the shocked samples. Each diffraction spot, which satisfied by the Bragg’s law, corresponded to an individual grain. At *t* = −3 ns, a few Bragg spots shifted to high Q side, whereas others did not move, corresponding, respectively to crystal volumes affected by the shock front and those in an uncompressed region.

The peak shifts of the diffraction spots, as shown in the magnification in Fig. 4, indicate that uniaxial shock compression of the individual grains behind the shock front took place. The elastically deformed grains were located just behind the shock wave front. This shift results from the elastic compression of one grain by an elastic precursor shock wave^19^. Subsequently, the plastic shock wave mainly deformed and reduced the size of each aluminum grain of on sub-nanosecond time scale after 0 ns^36^.

As the shock wave propagates into the aluminum sample, at *t* = 0 ns and 3 ns, azimuthally broadened diffraction spots evolve and the original spots begin to disappear. Broaden of the diffraction spots occurred from plastic deformation processes under shock-induced shearing and twisting, such as the induction of dislocations and dislocation networks, the formation of subgrains, grain refinement and (sub-)grain rotation. Notably, the spottiness of the rings transforms to a smooth dispersion at *t* = 6 ns, revealing grain refinement to less than 1 *μ*m^26,27^. A plastic shock flow follows the elastic shock wave, generating many defects and dislocations inside the shock-compressed region. The appearance of new broadened diffraction spots and the disappearance of the original sharp diffraction spots, and the ultimate appearance of a continuous Debye-Scherrer ring, indicates that plastic deformation occurs inside aluminum grains in the plastic shock flow. The appearance of new diffraction spots from the fractured small grains indicated dynamic grain rotation due to grains shearing and twisting in the plastic shock flow. These small grains, which originally have reciprocal lattice vectors that lie off the Evald sphere, move into the Laue condition^26^ and diffract X-rays under plastic shock wave loading. Grain rotation by thermomechanical processes into the preferred orientation at conventional, slow deformation rates is inherent to plastic deformation^9,26,27,37^. Similarly at high strain rates, ultrasmall grains are produced when the shock wave propagates through the large mother grain, rotating them under further plastic shock flow.

## Discussion

**Figure 5:**
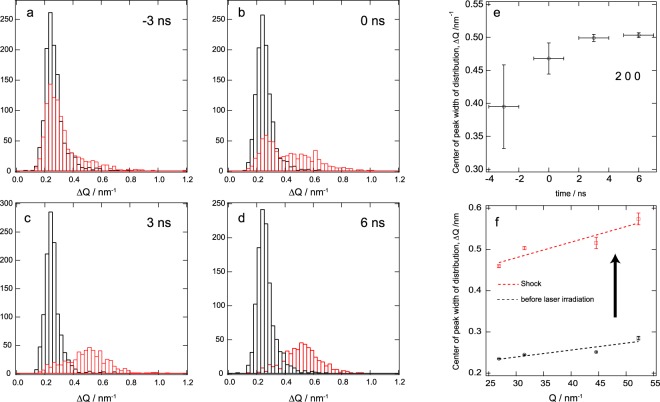
Williamson-Hall analysis of shock compressed polycrystalline aluminum. (**a**–**d**) Width distribution of the Bragg diffraction peak of the 111 aluminum reflection along the radial direction at ambient pressure (black curve) and at delay time of −3, 0, 3 and 6 ns (red curves). All data points of the Bragg diffraction spots were obtained from the average of 25 diffraction images. The Bragg diffraction spots broaden with shock wave loading. (**e**) Time-evolution of the center of the peak width distribution of the 200 reflection. (**f**) Williamson-Hall plot of aluminum during shock compression at 6 ns. The dotted line was fitted to the shock compression data using Eq. (3).

Figure 5a–d show the obtained histograms of the full width at half maximum (FWHM) for the reflection spots of aluminum 200 reflection at different delay times. The distribution shifted and uniformly broadened with shock wave propagation. The number of broadened peaks increased as the shock wave propagated through the polycrystalline aluminum foil. We counted approximately 1300 individual diffraction spots on each Debye-Scherrer ring at *G*_*hkl*_, which were acquired from 25 different diffraction images before and under shock compression. The diffraction spots were fitted by a Gaussian function to acquire a FWHM of Δ*Q* = Δ*G*_*hkl*_ at *G*_*hkl*_. The peak shift of the histogram also shifted and saturated at 3 ns because the shock wave had already arrived at the back surface, as shown in Fig. 5e. The stress rise time is related to the dislocation density and time scale on which dislocations grow with grain refinement and undergo rotation under plastic shock wave loading. For shock rise times on the order of a few nanoseconds, the width of the shock front became shorter than the aluminum grain sizes (i.e., a few ten to hundred micrometers), and complex shock wave interactions with grain boundaries arose together with inclusions, and crystal anisotropy. Such complexity might induce microstructure deformation of the polycrystalline metal.

We estimated the coherent grain size *D* and the inhomogeneous lattice strain $\epsilon$ under shock wave loading from the radial-width distribution of the diffraction spots. On the basis of the Williamson-Hall (W-H) approach^38^ and an expression in reciprocal space^39^, the total line width Δ*Q* comprised three sample-dependent terms: the Fourier transform of the coherent crystal size along $G\in Q$, which is equal to $K\cdot 2\pi$/*D*, where *K* is a shape factor equal to *K* = 0.94 for spherical particles; a strain-broadening term, $|\epsilon |$ = Δ*G*/*G*; and an instrument-broadening term, Δ${Q}_{inst}(Q)$. We define $G=2\pi$/*d*, in contrast to d* = 1/*d* in^39^, where *d* is a lattice spacing. Therefore, the Scherrer equation can be rewritten as:

3 $${\rm{\Delta }}Q=K\frac{2\pi }{D}+\epsilon G+{\rm{\Delta }}{Q}_{inst}(Q).$$

Figure 5f shows the Williamson-Hall plot comprising the aluminum 111, 200, 220, and 311 reflections before and 6 ns after laser irradiation, at which time the first shock wave has propagated through the sample. We assumed that the instrumental-broadening term, Δ${Q}_{inst}(Q)$, reflects the broadening of diffraction from the large aluminum crystallites before shock compression. We subtracted each distribution peak before laser irradiation from each distribution at 6 ns. These values can be determined from the slope and y-intercept of the fitting line using Eq. (3). Upon compression, there is a considerable increase of Δ*Q* for all reflections, which can be fitted by Δ$Q=A+BQ$, with A = 0.18 ± 0.03 nm^−1^ and B = 0.0021 ± 0.00066, which gives [from Eq. (3)] values of *D* = 33 nm and $\epsilon$ = 0.21 × 10^−2^. These result indicate that grains of polycrystalline material behind the shock wave decreased considerably from the micro- to nanometer scale. The shock recovery sample also showed grain refinement and many twins inside grain (Supplementary Fig. S1). The dislocation density ($\rho$) of the polycrystalline aluminum under shock wave loading, which is related to the plastic deformation, can be defined in term of the microstrain and grain size^38,40^,

4 $$\rho =\frac{2\sqrt{3}\epsilon }{Db}$$

where *b* is the magnitude of the Burgers vector for dislocation (0.286 nm for aluminum). Diffraction peak-broadening analysis based on the Williamson-Hall plot yields a dislocation density of 0.77 × 10^15^ m^−2^. Thus, the shock-induced deformation results in a microstructure that consists of ultrafine, nanometer-sized small grains with an increased dislocation density. The estimated dislocation density are 10^2^ to 10^4^ times lower than those of molecular dynamics simulations with face-centered cubic metals under shock wave loading^36^. However, the value of estimated dislocation density of shocked polycrystalline aluminum is consistent with the Meyers model (Supplementary Fig. S2), which was included with the dislocation moving at the shear wave velocity^8^. So far, in shock recovery experiments, the shock induced dislocation density observed by *ex*-*situ* experiments was low for the model. This previous result indicated that shock recovery experiments have shown that annihilation of shock induced dislocation occur during releasing the shock pressure.

Our observation demonstrated the possibility of studying the grain refinement process of materials under shock compression *in situ* by time-resolved synchrotron X-ray diffraction. Although laser-induced shock deformation has been applied industrially for laser peening, it remains difficult to gain *in situ* mechanistic insight into grain refinement and the increase in surface hardness. We directly observed grain refinement and the structural changes of polycrystalline metal under shock wave loading. Our technique will be valuable for revealing the mechanism of microstructural change of various alloy and ceramics materials based on dynamic processes.

The *in situ* crystalline size, microstrain, and dislocation density of polycrystalline aluminum can be determined from number of the spots in the Debye-Scherrer ring. Behind the shock wave front, large grains were compressed elastically along the shock wave direction. Ultrasmall grains of aluminum with high dislocation densities were generated under the subsequent plastic shock flow, and grains of aluminum were rotated owing to this plastic deformation. A number of ultra-fine grains were produced in the polycrystalline aluminum foil with a high dislocation density, which increased with the propagation of the laser-driven shock wave. Overall, these finding demonstrate the ability to study microstructural deformation in plastic shock flows from the atomic to mesoscale level.

## Methods

### Time-resolved X-ray diffraction

Measurements were all performed in a single shot pump-probe mode using a Nd:YAG laser (Powerlite 8000 Pro; Continuum Inc., CA, USA), which was operated at a wavelength of 1064 nm with a pulse width of 8 ns to to produced a shock wave into the sample. The laser spot size on the aluminum surface was 0.5 mm in diameter. The external laser trigger from the synchrotron radio-frequency master clock was reduced from 508 MHz to 9.46 Hz using frequency dividers. A detailed description of the timing system for the laser and X-ray pulse is given in refs ^19,30^. Timing jitter between the laser and X-ray pulse was about 1 ns. The laser intensity was 0.8 J/pulse. The default X-ray pulse from an undulator with a period length of 20 mm were 100 ps, 10^9^ photons/pulse, 15.61 keV, *λ* = 0.07949 nm, and *k* = 79.04 nm^−1^ respectively. X-ray was a 15% energy bandwidth of an asymmetric spectrum. For further monochromatization, the energy bandwidth and spectral shape were adjusted, such that the pulse possessed a symmetrical Gaussian shape of Δ*k*/*k* = 1.46% using an X-ray multilayer optics^41^, where the focused spot size of the X-ray pulse at the aluminum surface was 450 × 250 *μ*m^2^. A two-dimensional Debye-Scherrer ring was recorded for each delay-time between the laser-pump and the X-ray pulse, using a 165-mm-diameter charge-coupled device camera (MarCCD165, Rayonix Inc., IL, USA). A one-dimensional diffractogram was then created by integrating the detected Debye-Scherrer ring pattern along the azimuthal angle. A fresh area on the sample was used for each shot because the laser-irradiated area was completely destroyed after exposure of one laser shot. Finally, the laser beam direction was inclined by 15° from the X-ray path. The diffraction data was expressed against the modulus of the scattering vector *Q* = 2*k *sin(*θ*), where 2*θ* is the scattering angle, and the specific values $G\in Q$ denote the reciprocal-lattice points: that is, the peak position, G_*hkl*_, for a given reflection with Miller indices *hkl*.

### Shock-wave profile measurement using the VISAR system

The free-surface velocity of the polycrystalline aluminum film was recorded with a VISAR system on the rear side of the sample at the moment of shock release. The sample and laser irradiation conditions were same as those used for time-resolved X-ray diffraction. The VISAR system had a streak camera, which was synchronized with the shock-drive laser. The exact position of the VISAR system has adjusted with respect to the spot of shock-drive laser. Shock steadiness and shock planarity were guaranteed for the area that was irradiated by the X-ray pulse. The average pressure estimated from the VISAR system was in agreement with that estimated using the time-resolved X-ray diffraction method.

## Supplementary information

Supplementary info
