# Supplementary material for: Microstructural deformation process of shock-compressed polycrystalline aluminum
Source: Sci Rep. 2019 May 20;9:7604. doi: 10.1038/s41598-019-43876-2 (PMC6527857; doi:10.1038/s41598-019-43876-2)
Supplement: Supplementary file 1 — Supplementary info [file 41598_2019_43876_MOESM1_ESM.pdf]

Supplementary materials for

Microstructural deformation process of shock-compressed polycrystalline aluminum

Kouhei Ichiyanagi[1,2*], Sota Takagi[2,3], Nobuaki Kawai[4], Ryo Fukaya[2], Shunsuke Nozawa[2], Kazutaka G. Nakamura[5], Klaus-Dieter Liss[6,7], Masao Kimura[2], and Shin-ichi Adachi[2]

1 Division of Biophysics, Department of Physiology, Jichi Medical University, 3311-1 Yakushiji, Shimotuske, Tochigi 329-0498, Japan.

2 Photon Factory, Institute of Materials Structure Science, High Energy Accelerator Research Organization, 1-1 Oho, Tsukuba, Ibaraki 305-0801, Japan.

3 Division of Earth Evolution Science, Graduate School of Life and Environmental Sciences, University of Tsukuba, 1-1-1 Tennodai, Tsukuba, Ibaraki 305-8572, Japan.

4 Institute of Pulsed Power Science, Kumamoto University, 2-39-1 Kurokami, Kumamoto 860-8555, Japan

5 Laboratory for Materials and Structures, Tokyo Institute of Technology, R3-10, 4259 Nagatsuta, Yokohama, Kanagawa 226-8503

6 Materials and Engineering Science Program, Guangdong Technion-Israel Institute of Technology, 241 Daxue Road, Jinping District, Shantou, Guangdong 515063, China.

7 Tehnion-Israel Institute of Technology, Haifa 32000, Israel.

Supplementary Materials Includes:
Figure S1, Figure S2.

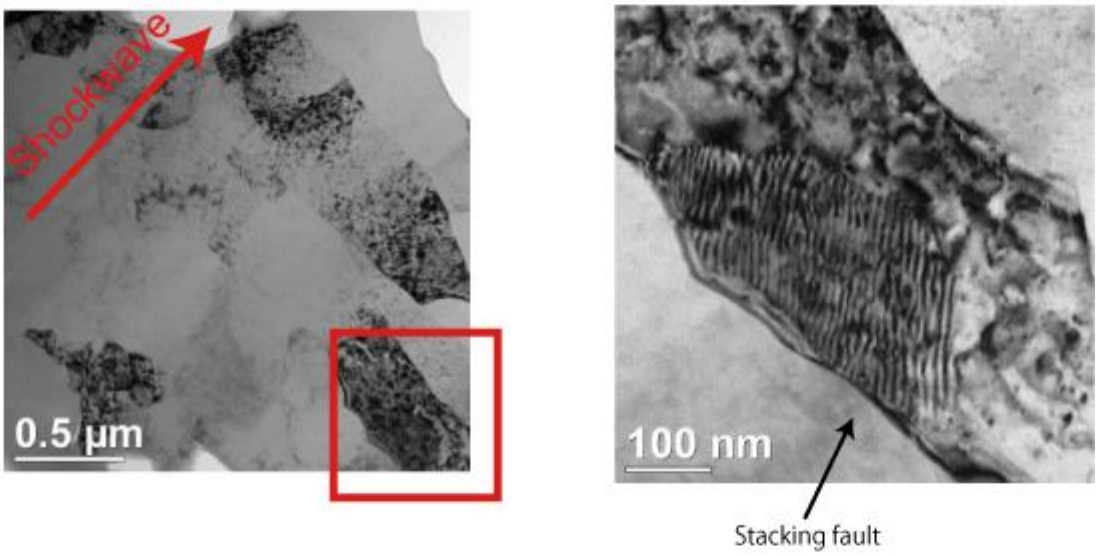

Fig. S1 Microstructure of shocked polycrystalline aluminum from 100-μm-thick sample examined by transmission electron microscope imaging. Microstructure of shock-compressed polycrystalline aluminum subjected to the same conditions as used for time-resolved X-ray diffraction. Grain size decreased and many twin lamellae appeared after shock-compression.

Estimation of dislocation density of shock compressed polycrystalline aluminum: Predicted dislocation densities of pure aluminum as a function of shock pressure are plotted in Fig. S2, and compared with actual results. If the dislocation is assumed to move at the shear wave velocity, the dislocation density can be expressed as a function of volume change, V/V_0[1]:

$$\rho = \left(\frac{0.4(1-\upsilon)\pi^2}{\sqrt{2}b^2}\right)\left(1-\left(\frac{V}{V_0}\right)^{1/3}\right)^3 \qquad (1)$$

where ν is Poisson's ratio and ρ is the dislocation density. b is the burgers vector of aluminum. Shock pressure, P can be expressed from the Rankine-Hugoniot equations and the equation of state:

$$\mathrm{P} = \frac{C_0^2\left(1-\frac{V}{V_0}\right)}{V_0\left[1-S\left(1-\frac{V}{V_0}\right)\right]^2} \qquad (1)$$

Our values for the dislocation density estimated from the diffraction peak width are

consistent with the calculated dislocation density.

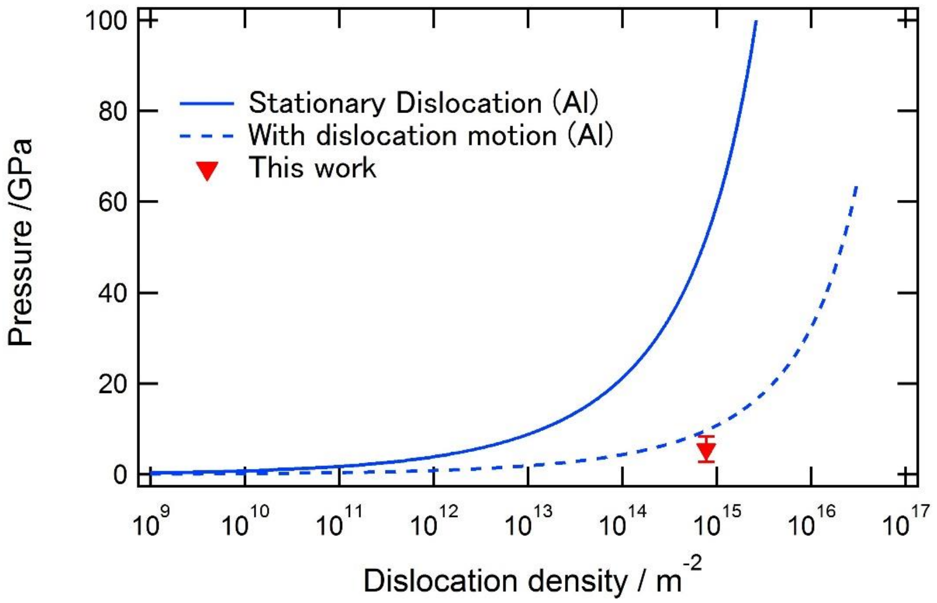

Fig. S2 Calculated dislocation densities in pure aluminum as a function of shock pressure. Variation of the dislocation density in aluminum, ρ with shock pressure P based on Eq. S1 and S2. The dotted curve shows the dislocation moving at the shear wave velocity. The solid curve shows the stationary dislocation under shock wave loading

[1] Meyers, M. A., Gregori, F., Kad, B. K., Schneider, M. S., Kalantar, D. H., Remington, B. A., Ravichandran, G., Boehly, T., & Wark, J. S., Laser-induced shock compression of monocrystalline copper: characterization and analysis. *Acta Materialia* **51**, 1211 (2003).
